# Changes in resting state functional connectivity after repetitive transcranial direct current stimulation applied to motor cortex in fibromyalgia patients

**DOI:** 10.1186/s13075-016-0934-0

**Published:** 2016-02-03

**Authors:** Chelsea M. Cummiford, Thiago D. Nascimento, Bradley R. Foerster, Daniel J. Clauw, Jon-Kar Zubieta, Richard E. Harris, Alexandre F. DaSilva

**Affiliations:** Neuroscience Graduate Program, University of Michigan, Ann Arbor, MI USA; Chronic Pain and Fatigue Research Center, Department of Anesthesiology, University of Michigan, Ann Arbor, MI USA; Headache & Orofacial Pain Effort (H.O.P.E.) Lab, Department of Biologic and Materials Sciences, School of Dentistry, University of Michigan, Ann Arbor, MI USA; Michigan Center for Oral Health Research, Michigan Institute for Clinical & Health Research, University of Michigan, Ann Arbor, MI USA; Ann Arbor VA Healthcare System, Ann Arbor, MI USA; Russell H. Morgan Department of Radiology, Johns Hopkins University, Baltimore, MD USA; Department of Radiology, University of Michigan, Ann Arbor, MI USA; Division of Rheumatology, Department of Internal Medicine, University of Michigan, Ann Arbor, MI USA; University Neuropsychiatric Institute, Department of Psychiatry, University of Utah Health Sciences Center, Salt Lake City, UT USA

**Keywords:** Repetitive transcranial direct current stimulation (tDCS), Fibromyalgia, Functional connectivity, Resting state, Motor cortex, fMRI

## Abstract

**Background:**

Fibromyalgia (FM) is a chronic, centralized pain condition characterized by alterations in the functional, chemical, and structural brain networks responsible for sensory and mood processing. Transcranial direct current stimulation (tDCS) has emerged as a potential treatment for FM. tDCS can alter functional connectivity (FC) in brain regions underneath and distant to the stimulating electrode, although the analgesic mechanisms of repetitive tDCS remain unknown. The aim of this study was to investigate how a clinically relevant schedule of tDCS sessions alters resting state FC and how these changes might relate to clinical pain.

**Methods:**

Resting state functional magnetic resonance imaging data were collected from 12 patients with FM at baseline, after 5 days of sham treatment, and after 5 days of real tDCS with the anode over the left primary motor cortex (M1) and the cathode over the right supraorbital cortex. Seed to whole-brain FC analyses were performed with seed regions placed in bilateral M1, primary somatosensory cortices (S1), ventral lateral (VL) and ventral posterolateral (VPL) thalami, and periaqueductal gray (PAG).

**Results:**

Stronger baseline FC between M1–VL thalamus, S1–anterior insula, and VL thalamus–PAG predicted greater analgesia after sham and real tDCS. Sham treatment (compared with baseline) reduced FC between the VPL thalamus, S1, and the amygdala. Real tDCS (compared with sham treatment) reduced FC between the VL thalamus, medial prefrontal, and supplementary motor cortices. Interestingly, decreased FC between the VL/VPL thalamus and posterior insula, M1, and S1 correlated with reductions in clinical pain after both sham and active treatments.

**Conclusions:**

These results suggest that while there may be a placebo response common to both sham and real tDCS, repetitive M1 tDCS causes distinct changes in FC that last beyond the stimulation period and may produce analgesia by altering thalamic connectivity.

**Electronic supplementary material:**

The online version of this article (doi:10.1186/s13075-016-0934-0) contains supplementary material, which is available to authorized users.

## Background

Fibromyalgia (FM) is a chronic centralized pain condition characterized by widespread pain, fatigue, sleep problems, cognitive dysfunction, and mood disturbances [1]. While the exact pathophysiology of FM remains unknown, a prevailing hypothesis states that a sensory processing dysfunction within the central nervous system creates, amplifies, or sustains the perception of chronic pain [2]. In support of this hypothesis, brain network alterations seen in these patients fall into two broad categories: decreased descending antinociceptive transmission, and/or enhanced pronociceptive processing [3–6].

Motor cortical dysfunction has been suggested in a number of chronic pain conditions, including FM. In general, the primary motor cortex (M1) shows increased cortical excitability at baseline and heightened responses to sensory stimuli, which may be suggestive of a reduction in inhibitory activity [7]. Noninvasive brain stimulation has emerged as an attractive therapeutic option for chronic pain conditions, given its ability to target specific cortical regions. Researchers in some studies have reported that transcranial direct current stimulation (tDCS) over M1 relieves pain in FM [8–10]. However, the authors of a recent review did not find a significant difference between sham and real M1 tDCS on short-term pain relief [11]. The lack of effect may be due to significant heterogeneity between the studies (i.e., stimulation parameters, number of treatment sessions, type of chronic pain) included in the review. It is also possible that sham tDCS produces a significant placebo response. Consistent with previous work implicating the endogenous opioid system in placebo analgesia [12, 13], we recently showed that sham tDCS caused the release of endogenous opioids in the periaqueductal gray (PAG), precuneus, and thalamus [14].

While placebo responses are clearly present in tDCS, the specific neurobiology underlying the analgesic effects of real tDCS are less clear. During and immediately after stimulation, tDCS may alter excitability by modulating resting membrane potential. Longer-lasting effects may be due to changes in synaptic plasticity via mechanisms similar to long-term potentiation or depression [15]. M1 tDCS can alter the functional connectivity (FC) of regions under the stimulating electrode [16], as well as spatially distant but structurally connected regions, such as the thalamus [17] and dorsolateral prefrontal cortex [18, 19]. Real tDCS also acts on the endogenous opioid system [14] similarly to invasive motor cortex stimulation (MCS) [20, 21]. However, these studies were conducted in healthy participants and the investigators examined FC during or shortly after M1 tDCS. There have been no investigations of how M1 tDCS alters resting state FC in patients with chronic pain treated repeatedly, as they might be in clinical practice.

We measured clinical pain and resting state FC in 12 patients with FM at baseline, after 5 days of sham treatment, and after 5 days of real tDCS. We were interested in three questions: (1) Does baseline connectivity predict clinical treatment response? (2) Are there differences in FC after sham and real tDCS? (3) Do changes in FC relate to analgesia? We hypothesized that strong M1–thalamus connectivity at baseline would predict a better clinical response, as shown in previous M1 stimulation studies [22, 23]. In addition, because we found a trend toward decreased glutamate + glutamine (Glx) in the thalamus after real tDCS in these same patients [24], and given the strong structural connectivity between M1 and the thalamus [25], we hypothesized that real tDCS would decrease FC between the thalamus and brain regions involved in pain perception.

## Methods

### Patients

We recruited 13 female patients with FM (age range 27–64 years, mean ± standard deviation 47.6 ± 10.6 years) for this study. One patient dropped out after the baseline visit, and the remaining twelve patients completed the entire protocol. All patients met the 1990 American College of Rheumatology criteria for FM [1], had experienced symptoms for at least 1 year, and reported pain on more than 50 % of days. The inclusion criteria were right-handed, a body mass index of 36 or less, and agreement to delay taking new medications or treatments for FM during the study. The exclusion criteria were pregnant or breastfeeding, participation in other clinical trials, currently taking opiates, history of autoimmune or chronic inflammatory disease that causes pain, substance abuse or severe psychiatric illness, and contraindications for magnetic resonance imaging procedures. The University of Michigan Institutional Review Board approved this study, and all subjects gave written informed consent. The effect of tDCS on brain metabolites in these same subjects is described in a previous report [24].

### Study design

Our within-subjects crossover design had three phases (Additional file 1: Figure S1). Session 1 included a baseline pain assessment and functional magnetic resonance imaging (fMRI). Session 2 consisted of sham tDCS for 5 consecutive days followed by pain assessment and a second fMRI. Session 3 comprised real tDCS for 5 consecutive days followed by pain assessment and a third fMRI 3. The sham and real tDCS phases were separated by a 7- to 11-day washout period (mean 9.9 days). We chose to perform real tDCS for 5 consecutive days because previous studies in patients with FM have shown a meaningful reduction in clinical pain using this protocol [8, 9]. To limit carryover from real to sham tDCS, we did not use a randomized design [26]. All participants were debriefed during a final follow-up visit. Patients were also offered a clinical referral to an outpatient clinic at our institution for continuation of care with regular therapy for their symptoms.

### Clinical pain outcomes

Clinical pain intensity was assessed as an “average” experience for the week before each assessment using a visual analogue scale (VAS), with 0 being “no pain” and 10 being “worst possible pain.” Clinical pain was also assessed using the short-form McGill Pain Questionnaire [27], and affective state was measured using the Positive and Negative Affect Schedule (PANAS) [28]. We were missing McGill baseline pain data for one patient, PANAS scores across all conditions for one patient, and PANAS baseline-only scores for two patients. The clinical results have been published previously [24] and are provided in Additional file 1: Table S1. Differences in clinical variables across conditions were assessed with repeated-measures analysis of variance (ANOVA) using IBM SPSS version 22 software (IBM, Armonk, NY, USA). Significance was set at an α level of *p* < 0.05. The changes in clinical pain scores used in neuroimaging analyses were calculated by subtraction of sham − baseline VAS and real − sham VAS.

### tDCS protocol

The tDCS protocol was carried out as described previously [29]. Briefly, for both sham and real tDCS sessions, the anode electrode was placed on the scalp over the left motor cortex and the cathode was positioned over the right supraorbital cortex. Positions were determined individually using the 10–20 international system of electroencephalogram electrode placement at C3 and FP2, respectively. Electrodes were placed by the same operators (AFD and TDN) for all patients. Active stimulation consisted of 2 mA of current applied continuously for 20 minutes. During sham tDCS, the current was applied for 30 seconds at the beginning and end of the session. Patients were blinded to type of treatment (i.e., real vs. sham) they were receiving. This protocol is identical to that used in previous studies of M1 tDCS in patients with FM [8, 9].

### Neuroimaging methods

Resting state fMRI sessions were performed on an Ingenia 3.0 T system (Philips Medical Systems, Best, the Netherlands) with a 15-channel receive head coil. Each scan lasted 10 minutes, and parameters included a T2*-weighted blood oxygen level–dependent (BOLD) echo-planar imaging sequence [repetition time (TR) 2000 ms, echo time (TE) 30 ms, flip angle 77 degrees, 30 slices, voxel size 3.44 × 3.33 × 4.00 mm]. Physiological data (cardiac and respiratory volume) were collected simultaneously. A high-resolution structural image was acquired for normalization purposes (TR/TE 9.8/4.6 ms, flip angle 8 degrees, 151 slices, voxel size 1 × 1 × 1 mm). fMRI data were checked for quality and head motion greater than 3 mm; no data were excluded. Resting state fMRI data were preprocessed using SPM8 (http://www.fil.ion.ucl.ac.uk/spm/software/spm8/) running on MATLAB R2010a (MathWorks, Natick, MA, USA) and included physiological artifact correction, slice timing correction, realignment, coregistration, normalization to Montreal Neurological Institute space, and smoothing (full width at half maximum 8 mm).

Seed to whole-brain FC analyses were performed using the Conn Toolbox [30]. Seeds were chosen on the basis of the following criteria: (1) location under stimulating anode (left precentral gyrus and left postcentral gyrus [M1/primary somatosensory cortex (S1); WFU PickAtlas (http://www.nitrc.org/projects/wfu_pickatlas)], (2) structural connectivity to left M1 and S1 [right pre- and postcentral gyri, bilateral ventral lateral (VL) and ventral posterolateral (VPL) thalamus; WFU PickAtlas), and (3) our previous tDCS studies (PAG [14]). The time series for each seed region was extracted and white matter, cerebrospinal fluid signal, and realignment parameters were entered into the analysis as regressors of no interest. A band-pass filter (0.008–0.09 Hz) was applied to remove linear drift artifacts and high-frequency noise. First-level analyses were performed by correlating the time series from each seed region with the rest of the voxels in the brain, creating seed to whole-brain Fisher-transformed correlation maps. These maps were imported into SPM8 for group-level analyses.

For prediction analyses, we performed seed to whole-brain regression analyses with baseline FC maps and change in clinical pain (real − baseline) as a regressor of interest. Main effects were calculated using repeated-measures ANOVA design with baseline, sham, and real tDCS FC maps. The contrasts of interest were baseline versus sham tDCS and sham versus real tDCS. We also examined the change in FC across the entire study using the contrast baseline versus real tDCS. To examine correlations between changes in connectivity and changes in clinical pain, we first created difference images by subtracting first-level connectivity maps for each subject (sham − baseline, real − sham, and real − baseline). We then performed a regression analysis with VAS change scores as a regressor of interest. All analyses were controlled for differences in age. Results were thresholded at uncorrected *p* < 0.001 on the voxel level and *p* < 0.05 familywise error (FWE) correction for multiple comparisons at the cluster level with a cluster size of greater than 5 voxels. For a priori regions that did not meet this stringent threshold, we performed small volume corrections (SVCs) using the anatomically (WFU PickAtlas) defined regions of interest (ROIs) used as seed regions or functionally defined ROIs derived from our previous findings in FM [6]. Significance for SVC was set at *p* < 0.05 FWE at the cluster level with a cluster size of greater than 5 voxels. The Fisher-transformed correlation values were extracted using MarsBaR software (http://marsbar.sourceforge.net/) and post hoc analyses performed using IBM SPSS version 22 software.

## Results

### Clinical pain reduction with sham and real tDCS

As reported previously [24], there was a trend toward improvement in VAS clinical pain during the sham period [mean difference ± standard error (SE) for sham minus baseline −1.042 ± 0.572, 95 % confidence interval (CI) −0.218 to 2.301; *p* = 0.096], and there was no significant difference in pain relief between sham and real tDCS (mean difference ± SE for real minus sham −0.750 ± 0.494, 95 % CI −1.838 to 0.338; *p* = 0.157). However, clinical pain significantly decreased across the entire study from baseline to after real tDCS (mean difference ± SE for real minus baseline −1.792 ± 0.762, 95 % CI −3.470 to −0.114; *p* = 0.038). There were no significant differences in clinical pain as measured by the McGill Pain questionnaire or PANAS positive affect. There was a significant difference between baseline and real tDCS in PANAS negative affect (mean difference ± SE for real minus baseline −3.0 ± 1.067, 95 % CI −5.461 to −0.539; *p* = 0.023). Clinical results for each patient individually across the study are presented in Additional file 1: Table S2.

### Stronger baseline FC is associated with subsequent analgesia

**Fig. 1:**
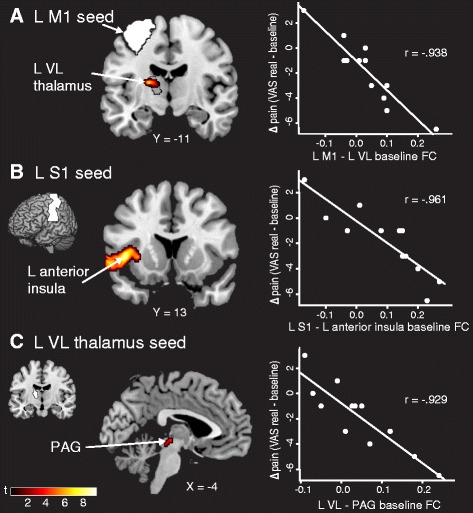
Stronger FC at baseline predicts analgesia. **a** Patients with higher L M1 (seed in *white*) − L VL (anatomical region outlined in *black*) connectivity at baseline had a greater reduction in clinical pain across sham and real transcranial direct current stimulation (tDCS) periods (displayed at *p* = 0.005). **b** Stronger L S1 (seed in *white*) − L anterior insula FC at baseline predicted a better clinical response. **c** Connectivity between the L VL thalamus (seed in *white*) and the PAG at baseline also predicted patients who would respond to sham and real tDCS treatment. Data shown are Fisher-transformed *r* values. *M1* primary motor cortex, *VL* ventral lateral, *S1* primary somatosensory cortex, *PAG* periaqueductal gray, *VAS* visual analogue scale, *L* left, *R* right, *FC* functional connectivity

To examine the common predictive ability of baseline FC for reductions in pain across sham and real tDCS (as this was where the significant clinical effect on pain was found), we used predefined ROIs and correlated baseline FC with improvements in clinical pain across the entire study period (real − baseline). Patients who had stronger connectivity at baseline between the left M1 seed and left VL thalamus (*p* = 0.011 FWE, SVC), between the left S1 seed and left anterior insula (*p* = 0.001 FWE), and between the left VL thalamus seed and PAG (*p* = 0.007 FWE, SVC) had greater improvement in clinical pain scores across sham and real tDCS periods (Fig. 1 and Table 1). Importantly, these correlations were also significant when we looked at change in clinical pain from baseline to sham or from sham to real tDCS alone (with one exception: left VL–PAG baseline FC and real–sham clinical pain; *p* = 0.057) (Additional file 1: Table S3). There were no regions that showed significant correlations between less connectivity at baseline and better treatment response (Additional file 1: Figure S2). In post hoc analyses, there were no significant correlations between baseline connectivity of these regions and the change McGill clinical pain or the change in positive and negative affect.

**Table 1:** Predicting changes in clinical pain from baseline functional connectivity

Seed	MNI coordinates (*x*, *y*, *z*)			*r* Value	T	Cluster size	Cluster *p* value
FC region							
L M1							
L VL thalamus	−18	−14	12	−0.938	5.41	7	0.011 FWE*
L S1							
L anterior insula	−42	14	2	−0.961	9.38	396	0.001 FWE
L VL thalamus							
PAG	−6	−26	−8	−0.929	5.25	8	0.007 FWE*

*FC* functional connectivity, *L* left, *M1* primary motor cortex, *VL* ventral lateral, *S1* primary somatosensory cortex, *PAG* periaqueductal gray, *MNI* Montreal Neurological Institute *T* test statistic

*Significant at *p* < 0.05 with small volume correction

### Sham tDCS is associated with decreases in FC

**Fig. 2:**
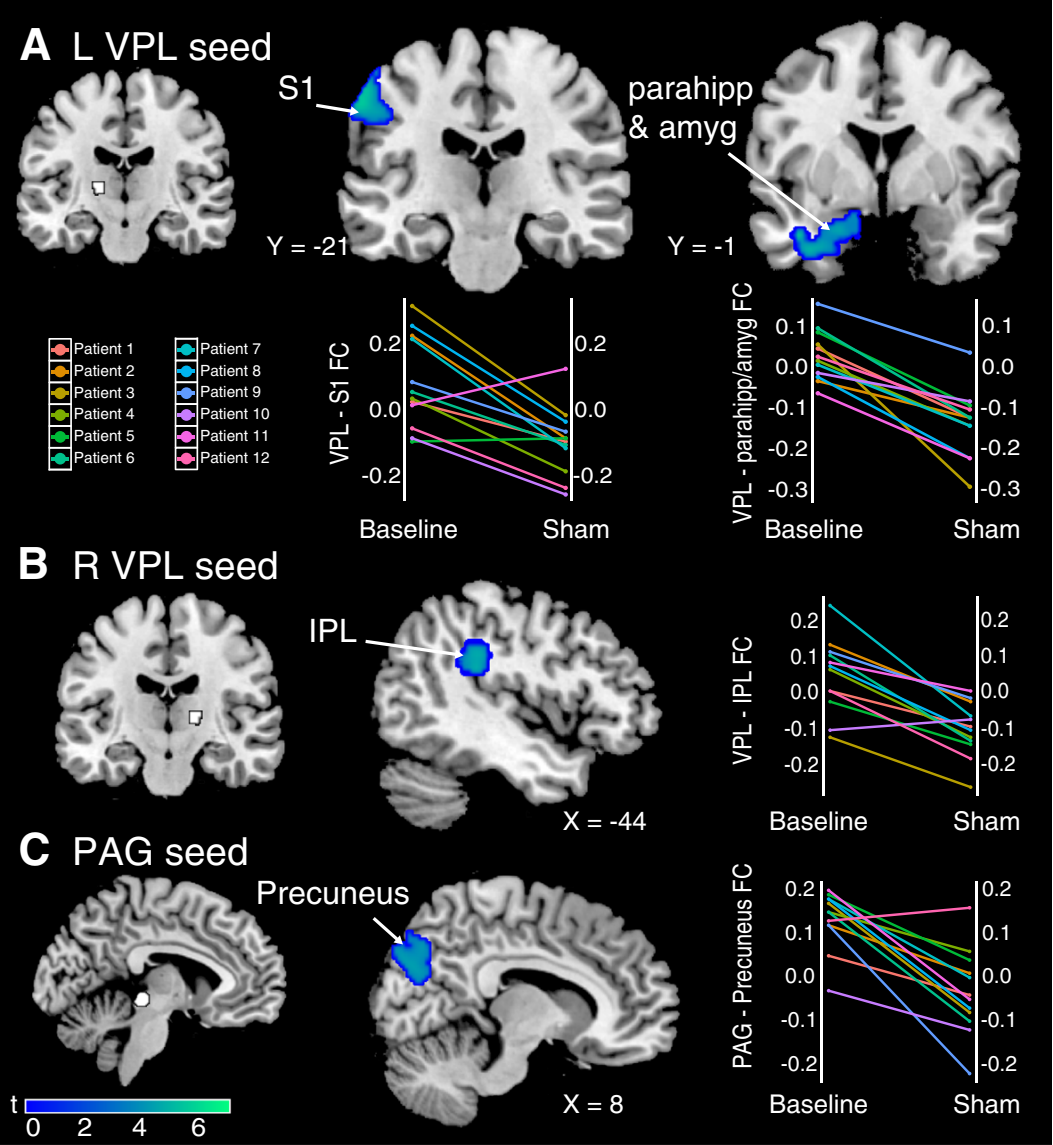
Sham transcranial direct current stimulation (tDCS) decreases FC compared with baseline. **a** Decreased connectivity between the left VPL (seed in *white*) and left S1, left parahippocampal gyrus, and amygdala after sham tDCS compared with baseline. Plots show changes in FC from baseline to after the sham treatment period for each patient with fibromyalgia. **b** Decreased connectivity between the right VPL (seed in *white*) and left IPL. **c** Decreased connectivity between the PAG (seed in *white*) and precuneus. Data shown are Fisher-transformed *r* values. *VPL* ventral posterolateral, *S1* primary somatosensory cortex, *parahipp* parahippocampal gyrus, *amyg* amygdala, *IPL* inferior parietal lobule, *PAG* periaqueductal gray, *L* left, *R* right, *FC* functional connectivity

Because previous studies have shown a placebo analgesic response on experimental and clinical pain during sham tDCS [31], we examined whether sham tDCS changed resting state FC (sham − baseline) (Table 2). After five sessions of sham tDCS, patients with FM had reduced FC between the left VPL thalamus seed and left S1 (*p* = 0.016 FWE), left amygdala/parahippocampal gyrus (*p* = 0.004 FWE), and right inferior parietal lobule (IPL) (*p* = 0.013 FWE) (Fig. 2a). FC also decreased between the right VPL thalamus seed and left IPL (*p* = 0.049 FWE) (Fig. 2b), between the PAG seed and precuneus (*p* = 0.001 FWE) (Fig. 2c), and between the right M1 seed and right cerebellum (*p* = 0.002 FWE). There were no significant increases in FC after sham compared with baseline (Additional file 1: Figure S3).

**Table 2:** Main effect of sham tDCS on FC

Seed	MNI coordinates (*x*, *y*, *z*)			T	Cluster size	Cluster *p* value
FC region						
Baseline > sham						
L VPL						
L S1	−62	−16	42	6.80	304	0.016 FWE
L parahipp/amyg	−32	−14	−26	6.70	408	0.004 FWE
R IPL	44	−36	32	5.69	320	0.013 FWE
R VPL						
L IPL	−34	−36	34	5.28	230	0.049 FWE
R M1 (precentral gyrus)						
R cerebellum	16	−74	−36	5.91	485	0.002 FWE
PAG						
Precuneus	−20	−84	24	4.88	618	0.001 FWE
Baseline < sham						
N.S.						

*tDCS* transcranial direct current stimulation, *FC* functional connectivity, *L* left, *R* right, *VPL* ventral posterolateral, *S1* primary somatosensory cortex, *parahipp/amyg* parahippocampal gyrus and amygdala, *IPL* inferior parietal lobule, *M1* primary motor cortex, *PAG* periaqueductal gray, *N.S*. not significant, *MNI* Montreal Neurological Institute *T* test statistic

**Fig. 3:**
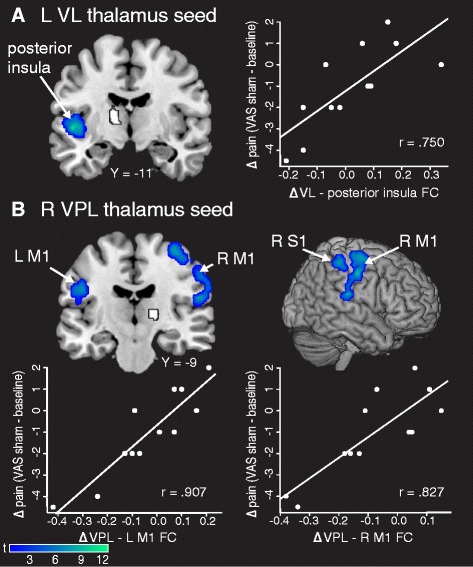
Correlations between changes in FC and changes in clinical pain after sham transcranial direct current stimulation (tDCS). **a** Decreased FC between the left VL thalamus (seed in *white*) and left posterior insula was correlated with a reduction in clinical pain after sham tDCS. **b** Decreased FC between the right VPL thalamus (seed in *white*) and left M1, right M1, and right S1 correlated with reduced clinical pain after sham tDCS. Data shown are Fisher-transformed *r* values. *VL* ventral lateral, *VPL* ventral posterolateral, *M1* primary motor cortex, *S1* primary somatosensory cortex, *VAS* visual analogue scale, *L* left, *R* right, *FC* functional connectivity

To determine if changes in FC were related to changes in clinical pain during the sham period, we ran a regression analysis with each participant’s connectivity difference map (sham − baseline) with change in VAS (sham − baseline) as a regressor of interest (Table 3). The change in connectivity between the left VL thalamus seed and the left posterior insula was positively correlated with change in clinical pain (*p* = 0.001 FWE). Patients with reduced connectivity between the left VL thalamus and posterior insula had a greater reduction in pain intensity after sham tDCS (Fig. 3a). Reduced connectivity between the right VPL thalamus seed and right M1 (*p* = 0.001 FWE), right S1 (*p* = 0.008 FWE), and left M1 (*p* = 0.046 FWE) also correlated with reduced pain after sham tDCS (Fig. 3b). Decreased FC between the right S1 seed and the cerebellum (*p* = 0.001 FWE) was also positively correlated with change in pain. These changes in connectivity were not significantly correlated with changes in positive and negative affect or McGill clinical pain. There were no significant relationships between increases in connectivity and decreases in clinical pain (Additional file 1: Figure S6).

**Table 3:** Correlations between change in FC and change in clinical pain (VAS) for sham versus baseline

Seed	MNI coordinates (*x*, *y*, *z*)			*r* Value	T	Cluster size	Cluster *p* value
FC region							
L VL thalamus							
L posterior insula	−48	−12	0	0.979	12.33	313	0.001 FWE
R VPL thalamus							
R M1	56	−12	42	0.969	9.85	603	0.000 FWE
R S1	44	−34	54	0.917	7.46	235	0.008 FWE
L M1	−46	−6	26	0.936	9.41	158	0.046 FWE
R S1 (postcentral gyrus)							
Cerebellum	36	−52	−20	0.937	7.63	355	0.001 FWE

*FC* functional connectivity, *VAS* visual analog scale, *L* left, *R* right, *VL* ventral lateral, *VPL* ventral posterolateral, *M1* primary motor cortex, *S1* primary somatosensory cortex, *MNI* Montreal Neurological Institute *T* test statistic

### Real tDCS is also associated with decreases in FC

**Fig. 4:**
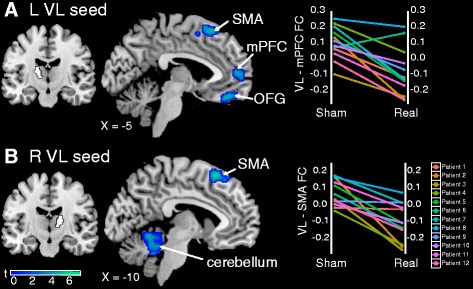
Real transcranial direct current stimulation (tDCS) decreases FC compared with sham. **a** Decreased connectivity between the left VL (seed in *white*) and SMA and mPFC after real tDCS. **b** Decreased connectivity between the right VL (seed in *white*) and SMA and cerebellum after real tDCS. Plots show changes in FC between sham and real tDCS for each patient with fibromyalgia. Data shown are Fisher-transformed *r* values. *OFG* orbitofrontal gyrus, *VL* ventral lateral, *SMA* supplementary motor area, *mPFC* medial prefrontal cortex, *L* left, *R* right, *FC* functional connectivity

Next, we measured changes in FC between sham and real tDCS (Table 4). After real tDCS, FC decreased between the left VL thalamus seed and the medial prefrontal cortex (mPFC) (*p* = 0.006 FWE) and left supplementary motor area (SMA) (*p* = 0.043 FWE) (Fig. 4a). FC also decreased between the right VL thalamus seed and the cerebellum (*p* = 0.001 FWE) and left SMA (*p* = 0.016 FWE) (Fig. 4b). There were no significant increases in FC (Additional file 1: Figure S3).

**Table 4:** Main effect of real tDCS on FC

Seed	MNI coordinates (*x*, *y*, *z*)			T	Cluster size	Cluster *p* value
FC region						
Sham > real						
L VL thalamus						
mPFC	4	56	8	5.66	362	0.006 FWE
L SMA	−2	24	56	5.49	228	0.043 FWE
L OFG	−10	40	−22	5.91	185	0.08 FWE^a^
R VL thalamus						
Cerebellum	16	−46	−22	6.88	1122	0.000 FWE
L SMA	−6	22	58	6.16	313	0.016 FWE
Sham < real						
N.S.						

*tDCS* transcranial direct current stimulation, *FC* functional connectivity, *L* left, *R* right, *VL* ventral lateral, *mPFC* medial prefrontal cortex, *SMA* supplementary motor area, *OFG* orbitofrontal gyrus, *N.S*. not significant, *MNI* Montreal Neurological Institute *T* test statistic

^a^Trend at *p* < 0.05 familywise error correction for multiple comparisons

When we compared baseline with real tDCS, we found significant decreases in connectivity between the left VPL thalamus seed and the left IPL (*p* = 0.041 FWE) and between the PAG seed and the posterior cingulate (*p* = 0.007 FWE) (Additional file 1: Figure S4 and Table S4). There were no significant increases in FC.

**Fig. 5:**
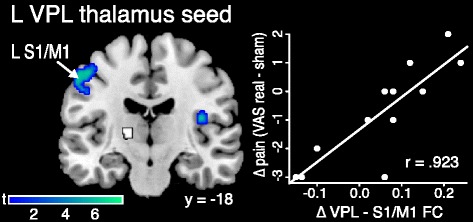
Correlation between change in FC and change in clinical pain after real transcranial direct current stimulation (tDCS). Patients with reduced FC between the L VPL thalamus (seed in *white*) and left S1/M1 and right posterior insula had greater reductions in clinical pain after real tDCS compared with sham (displayed at *p* = 0.005). Data shown are Fisher-transformed *r* values. *VPL* ventral posterolateral, *S1* primary somatosensory cortex, *M1* primary motor cortex, *VAS* visual analogue scale, *L* left, *R* right, *FC* functional connectivity

We did not find any regions that met whole-brain correction in a regression analysis measuring changes in connectivity in relation to changes in pain after real tDCS compared with sham. However, there were regions that met significance using SVC with *a priori* ROIs (Table 5). The change in connectivity between the left VPL thalamus and left M1/S1 (*p* = 0.007 FWE, SVC) and right posterior insula (*p* = 0.007 FWE, SVC) was positively correlated with the change in clinical pain (Fig. 5). The change in left VL thalamus to right posterior insula connectivity was also positively correlated with change in pain (*p* = 0.022 FWE, SVC). Patients with reduced connectivity between the VL/VPL thalamus and M1/S1 and posterior insula had a greater reduction in pain intensity after real tDCS. In post hoc analyses, these changes in connectivity were also correlated with the change in McGill clinical pain (Additional file 1: Table S6). However, there were no significant relationships between FC and changes in positive or negative affect. In an analysis in which we examined changes in connectivity and changes in clinical pain from baseline to real tDCS, we found that patients with reduced connectivity between left S1 and left SMA had a greater reduction in clinical pain (*p* = 0.013 FWE) (Additional file 1: Figure S5 and Table S5). Again, there were no significant relationships between increases in connectivity and decreases in clinical pain, neither between sham and real tDCS nor between baseline and real tDCS (Additional file 1: Figure S6).

**Table 5:** Correlations between change in FC and change in clinical pain (VAS) for real versus sham

Seed	MNI coordinates (*x*, *y*, *z*)			*r* Value	T	Cluster size	Cluster *p* value
FC region							
L VL thalamus							
R posterior insula	42	−24	20	0.887	5.08	5	0.022 FWE^a^
L VPL thalamus							
L M1/S1	−48	−18	50	0.923	6.45	89	0.007 FWE^a^
R posterior insula	46	−30	24	0.927	7.64	23	0.007 FWE^a^

*FC* functional connectivity, *VAS* visual analogue scale, *L* left, *R* right, *VL* ventral lateral, *VPL* ventral posterolateral, *M1* primary motor cortex, *S1* primary somatosensory cortex, *MNI* Montreal Neurological Institute *T* test statistic

^a^Significant at *p* < 0.05 with small volume correction

## Discussion

**Fig. 6:**
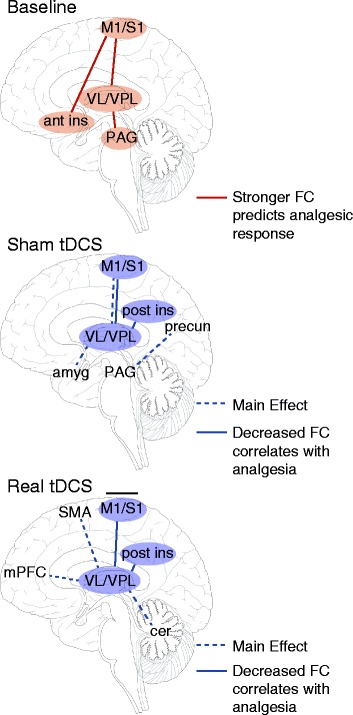
Summary of results. Stronger corticothalamic FC and FC between regions with high densities of opioid receptors at baseline predicted a better clinical response across the entire study. Changes in FC were observed after sham transcranial direct current stimulation (tDCS), which could be attributed to placebo analgesia or regression to the mean. Real tDCS caused some distinct long-lasting changes in FC (compared with sham) and may have relieved pain via the inhibition of thalamic activity and subsequent decreases in FC, both of which could have been caused by the release of endogenous opioids. *M1* primary motor cortex, *S1* primary somatosensory cortex, *VL* ventral lateral, *VPL* ventral posterolateral, *Ant Ins* anterior insula, *PAG* periaqueductal gray, *Post Ins* posterior insula, *Amyg* amygdala, *Precun* precuneus, *SMA* supplemental motor area, *mPFC* medial prefrontal cortex, *Cer* cerebellum, *FC* functional connectivity

This study shows, for the first time to our knowledge, that a clinically relevant schedule of repetitive M1 tDCS sessions alters FC in patients with FM. Real tDCS (versus sham) reduced FC between the VL thalamus and SMA, mPFC, and the cerebellum. These changes in FC were distinct from those observed after sham tDCS. Sham tDCS (compared with baseline) decreased connectivity between the VPL thalamus and S1, IPL, and the parahippocampal gyrus/amygdala and between the PAG and precuneus. However, after both sham and active tDCS, we found a relationship between decreases in FC among pronociceptive brain regions and reductions in clinical pain. Patients with decreased connectivity between the VL thalamus and posterior insula and between the VPL thalamus and M1/S1 had greater reductions in clinical pain after sham and real tDCS. In addition, our data indicate that patients with FM with stronger baseline connectivity between left M1 and left VL thalamus, between left S1 and left anterior insula, and between left VL thalamus and PAG had a better analgesic response across the entire study. Although we saw distinct main effects for sham and active tDCS, the overlapping results related to clinical changes in pain may point to a shared placebo response in both sham and active conditions. A summary of the results is depicted in Fig. 6.

Our findings are somewhat at odds with the existing literature. For example, researchers in some studies have reported increases in thalamic blood flow [32] or increased M1–thalamus connectivity after M1 stimulation [17, 33]. We suggest that these conflicting results can be explained by the timing of stimulation and measurement. In the other studies, neural activity was measured during or immediately after M1 stimulation, which likely has a different neural signature than after 1 week of repetitive stimulation. In support of this notion, García-Larrea and colleagues noted that thalamic blood flow reverted to baseline levels 30 minutes after M1 stimulation stopped [32]. Therefore, the initial or acute changes in thalamic activity may cause a cascade of other events that are important for analgesia [34], leading to the distinct long-term changes that we observed.

How might M1 stimulation promote analgesia in patients with chronic pain? One hypothesis states that M1 stimulation suppresses pain perception directly by inhibiting activity in the lateral thalamus [35, 36]. Compared with healthy control subjects, patients with FM have increased activity in pain-processing structures during experimental pain and increased connectivity in ascending pronociceptive pathways at rest (see [37] for a recent review on neuroimaging findings in FM), although the specific role of the thalamus in FM remains unclear. Both increases and decreases in thalamic activity or connectivity during experimental pain or at rest have been reported [3, 4, 38, 39]. In the present study, we found that strong M1–VL thalamus connectivity at baseline predicted a greater reduction in pain across sham and real tDCS periods. This is consistent with work in central poststroke pain, where analgesic response to repetitive transcranial magnetic stimulation over M1 was found to be best in patients with intact thalamocortical tracts (as measured by diffusion tensor imaging) [22, 23]. Invasive MCS decreases thalamic hyperactivity [40], likely by activating GABAergic divisions of the thalamus and increasing inhibition [41]. In healthy rodents and in a rodent model of neuropathic pain, MCS decreased the firing rate of VPL thalamic neurons specifically [42, 43]. In a previous study of the same patients reported here, we found a trend toward decreased Glx in the bilateral thalamus after real tDCS compared with sham [24]. The decreases in FC between the thalamus and SMA, mPFC, and cerebellum after real tDCS in this study lends support to the hypothesis that M1 stimulation disrupts thalamic activity.

Another hypothesis is that M1 stimulation causes analgesia indirectly via the facilitation of descending antinociceptive pathways and release of endogenous opioids [34]. Maarrawi and colleagues hypothesized that patients with neuropathic pain who have higher levels of endogenous opioids at baseline would be least likely to benefit from any additional opioid release caused by MCS, and, indeed, patients with lower baseline binding potential for an opioid agonist (reflecting either fewer opioid receptors or higher levels of endogenous opioids) in the thalamus, insula, and PAG were the least likely to benefit from MCS [20]. The relationship between opioids and BOLD fMRI signaling deserves further study, but in healthy controls morphine administration decreases activity in S1, thalamus, and PAG [44] while naloxone (an opioid antagonist) increases activity in S1, thalamus, insula, and PAG [45]. In our study, stronger M1–thalamus, S1–insula, and thalamus–PAG connectivity at baseline predicted a better treatment response. Because both sham and real tDCS also cause endogenous opioid release [14], this finding may suggest that patients with connectivity between regions under the stimulating anode (M1/S1) and regions with a high density of opioid receptors are the most likely to benefit from tDCS. We also found decreases in FC in many of these regions after sham and real tDCS, which could also reflect opioid release.

Placebo analgesia is a well-documented psychobiological event, and imaging studies have revealed overlap between brain networks involved in pain processing and those implicated in the placebo response [46, 47]. The decreases in FC found after sham (compared with baseline) are consistent with previous studies of placebo analgesia showing decreased activity in the thalamus, S1, amygdala, and insula [48–50]. Importantly, the similarity of FC results related to changes in clinical pain between baseline and sham and between sham and real sessions may suggest a shared placebo component across conditions. However, the changes in FC after the sham period in this study cannot be interpreted solely as a placebo effect, because we lacked a control group that received no treatment for comparison. Therefore, any changes from baseline to sham could also reflect regression to the mean or the natural course of the disease [51]. Regression to the mean also may account for a portion of the change in clinical pain after both the sham and real tDCS periods.

Our study was limited by the small sample size, which may have contributed to the lack of a significant clinical effect between sham and active conditions and reduced statistical power. In this pilot study, we did not aim to validate the efficacy of M1 tDCS as a treatment for FM; rather, our goal was to determine if a clinically relevant schedule of tDCS sessions altered resting state FC in patients with FM. However, as with most pain treatments wherein only a portion have a clinically meaningful response, half of the patients in the present study did report a drop in pain on the VAS (active − sham), and this change was correlated with reductions in thalamocortical connectivity. This study is significantly limited by the lack of counterbalancing between sham and active conditions. Therefore, we cannot rule out carryover effects from the sham period. However, sham and active phases were separated by a washout period of at least 1 week, so we find this unlikely. Another limitation is that the stimulation sessions were single-blinded. Finally, research has shown that stimulation for 30 seconds at the beginning and end of the sham condition can mimic the sensation during active treatment and that patients cannot differentiate between sham and real stimulation at 1 mA [52]. However, the credibility of sham tDCS and the effectiveness of patient blinding at 2 mA has recently been questioned [11]. Given our repeated-measures design, it is likely that the patients became aware of the condition differences. Our results should be interpreted with caution, and additional studies designed to replicate our findings are needed.

## Conclusions

Our results support the hypothesis that repetitive M1 tDCS causes lasting changes in FC and may relieve pain by altering thalamic activity. Analgesia may result from the inhibition of thalamic activity and subsequent decreases in FC, both of which could be caused by the release of endogenous opioids. Future studies that combine fMRI and positron emission tomography within the same patients after repetitive M1 tDCS are needed to test this hypothesis. It is possible that there is a significant placebo component common to both sham and real tDCS. Future studies should include a no-treatment control group to test this hypothesis. It remains to be seen if similar changes in FC are observed for tDCS in other chronic pain conditions.

## Additional file

Additional file 1: (DOCX 3033 kb)

## Abbreviations

ANOVA: analysis of variance
BOLD: blood oxygen level–dependent
CI: confidence interval
FC: functional connectivity
FM: fibromyalgia
fMRI: functional magnetic resonance imaging
FWE: familywise error
Glx: glutamate + glutamine
IPL: inferior parietal lobule
M1: primary motor cortex
MCS: motor cortex stimulation
MNI: Montreal Neurological Institute
mPFC: medial prefrontal cortex
N.S.: not significant
OFG: orbitofrontal gyrus
PAG: periaqueductal gray
PANAS: Positive and Negative Affect Schedule
ROI: region of interest
S1: primary somatosensory cortex
SE: standard error
SMA: supplementary motor area
SVC: small volume correction
tDCS: transcranial direct current stimulation
TR: repetition time
TE: echo time
VAS: visual analogue scale
VL: ventral lateral
VPL: ventral posterolateral
