# Supplementary material for: Changes in resting state functional connectivity after repetitive transcranial direct current stimulation applied to motor cortex in fibromyalgia patients
Source: Arthritis Res Ther. 2016 Feb 3;18:40. doi: 10.1186/s13075-016-0934-0 (PMC4741001; doi:10.1186/s13075-016-0934-0)
Supplement: Additional file 1: — (DOCX 3033 kb) [file 13075_2016_934_MOESM1_ESM.docx]

**Additional file 1**

**Figure S1:**

Figure S1 Legend:

Our within-subjects crossover design had three phases: a baseline pain assessment and functional magnetic resonance imaging (fMRI) session #1, sham tDCS for five consecutive days followed by pain assessment and fMRI #2, and real tDCS for five consecutive days followed by pain assessment and fMRI #3. Sham and real tDCS phases were separated by a 7-11 day washout period (mean = 9.9 days).

**Figure S2:**

Figure S2 Legend:

FM patients who had stronger FC at baseline between the left M1 seed and left VL thalamus, between left S1 and left anterior insula and between left VL thalamus and the PAG had greater improvement in clinical pain across sham and real tDCS. There were no regions that showed significant correlations between less FC at baseline and greater improvement in clinical pain. The glass brain results for the left M1 seed are depicted at a voxel threshold of p<0.001.

**Figure S3:**

Figure S3 Legend:

**A,** The glass brain results for the left VPL thalamus seed are depicted at a voxel threshold of p<0.001 for the baseline > sham and sham > baseline contrasts. Significant results were only found for baseline > sham (see Table 2 main text). **B,** The glass brain results for the left VLL thalamus seed are depicted at a voxel threshold of p<0.001 for the sham > real tDCS and real tDCS > sham contrasts. Significant results were only found for sham > real tDCS (see Table 4 main text).

**Figure S4:**

Figure S4 Legend:

Real tDCS decreases FC compared to baseline. **A,** Decreased connectivity between the left VPL (seed in white) and IPL after real tDCS. Plots show changes in FC between baseline and real tDCS for each FM patient. **B,** Decreased connectivity between the PAG (seed in white) and PCC after real tDCS. VPL, ventral posterior lateral; IPL, inferior parietal lobule; PAG, periaqueductal gray; PCC, posterior cingulate; L, left; R, right; FC, functional connectivity (fisher transformed r-values).

**Figure S5:**

Figure S5 Legend:

Correlation between change in FC and change in clinical pain after real tDCS. Patients with reduced FC between the left S1 (seed in white) and left SMA had greater reductions in clinical pain after real tDCS compared to baseline. S1, primary somatosensory cortex; SMA, supplementary motor area; VAS, visual analog scale; L, left; R, right; FC, functional connectivity (fisher transformed r-values).

**Figure S6:**

Figure S6 Legend:

**A,** The glass brain results for the right VPL thalamus seed are depicted at a voxel threshold of p<0.001. Significant results were only found for correlations between reductions in connectivity and reductions in clinical pain after sham tDCS (see Table 3 main text). There were no significant correlations between increases in connectivity and reductions in clinical pain. **B,** The glass brain results for the left VPL thalamus seed are depicted at a voxel threshold of p<0.001. Significant results were only found for correlations between reductions in connectivity and reductions in clinical pain after real tDCS (see Table 5 main text). There were no significant correlations between increases in connectivity and reductions in clinical pain.

**Table S1:**

Clinical Results

|  | Baseline (mean ± SD) | Sham (mean ± SD) | Real tDCS (mean ± SD) |
| --- | --- | --- | --- |
| Clinical Pain Intensity (VAS) | 5.12 ± 2.30 | 4.08 ± 2.11 | 3.33 ± 2.84* |
| McGill Total Pain | 24.09 ± 15.08 | 18.67 ± 12.47 | 19.33 ± 15.30 |
| PANAS (Positive Affect) | 19.78 ± 5.99 | 17.82 ± 5.47 | 16.09 ± 6.41 |
| PANAS (Negative Affect) | 14.33 ± 3.39 | 15.36 ± 5.46 | 12.73 ± 3.58* |

* Significantly different from baseline at p < 0.05

Table S2:

Patient Characteristics

|  | **Age** | **VAS Baseline** | **VAS Sham** | **VAS Real** | **McGill Baseline** | **McGill Sham** | **McGill Real** | **PANAS positive Baseline** | **PANAS positive Sham** | **PANAS positive Real** | **PANAS negative Baseline** | **PANAS negative Sham** | **PANAS negative Real** |
| --- | --- | --- | --- | --- | --- | --- | --- | --- | --- | --- | --- | --- | --- |
| Patient 1 | 34 | 3 | 5 | 2 | 11 | 18 | 14 | Missing data | Missing data | Missing data | Missing data | Missing data | Missing data |
| Patient 2 | 46 | 5 | 6 | 8 | 37 | 33 | 55 | 30 | 19 | 14 | 16 | 20 | 14 |
| Patient 3 | 54 | 8 | 6 | 5 | 27 | 13 | 23 | 14 | 10 | 10 | 21 | 27 | 10 |
| Patient 4 | 37 | 4 | 4 | 3 | 36 | 16 | 27 | 20 | 20 | 30 | 15 | 16 | 11 |
| Patient 5 | 64 | 0 | 0 | 0 | 0 | 0 | 0 | 16 | 20 | 22 | 13 | 10 | 10 |
| Patient 6 | 56 | 4 | 3 | 0 | 37 | 42 | 22 | 13 | 13 | 14 | 11 | 10 | 10 |
| Patient 7 | 58 | 6.5 | 2 | 0 | 8 | 4 | 0 | 19 | 26 | 14 | 14 | 13 | 12 |
| Patient 8 | 52 | 6 | 2 | 3 | 31 | 27 | 24 | 29 | 24 | 23 | 10 | 10 | 10 |
| Patient 9 | 54 | 4 | 3 | 3 | 10 | 11 | 10 | Missing data | 10 | 10 | Missing data | 16 | 20 |
| Patient 10 | 40 | 6 | 7 | 7 | Missing data | 12 | 27 | Missing data | 15 | 10 | Missing data | 18 | 18 |
| Patient 11 | 45 | 7 | 5 | 2 | 21 | 16 | 4 | 19 | 16 | 13 | 12 | 10 | 10 |
| Patient 12 | 52 | 8 | 6 | 7 | 47 | 32 | 26 | 18 | 23 | 17 | 17 | 19 | 15 |

**Table S3:**

Baseline FC Predicts Subsequent Analgesia

|  | Δ Clinical Pain (VAS) Sham - Baseline | Δ Clinical Pain (VAS)  Real tDCS - Sham | Δ Clinical Pain (VAS)  Real tDCS - Baseline |
| --- | --- | --- | --- |
| L M1 – L VL Baseline FC | r = -0.619  p = 0.042 | r = -0.791  p = 0.004 | r = -0.938  p = 0.001 |
| L S1 – L anterior insula Baseline FC | r = -0.671  p = 0.024 | r = -0.774  p = 0.005 | r = -0.961  p = 0.001 |
| L VL – PAG Baseline FC | r = -0.815  p = 0.002 | r = -0.587  p = 0.057 | r = -0.929  p = 0.001 |

FC, functional connectivity; L, left; M1, primary motor cortex; VL, ventral lateral; S1, primary somatosensory cortex; PAG, periaqueductal gray

**Table S4:**

Main effect of Real tDCS compared to Baseline

| Seed  FC Region | MNI coordinates (x y z) | | | T | Cluster size | Cluster p-value |
| --- | --- | --- | --- | --- | --- | --- |
| **Baseline > Real** |  |  |  |  |  |  |
| L VPL thalamus |  |  |  |  |  |  |
| L IPL | -46 | -50 | 34 | 5.40 | 239 | 0.041 FWE |
| PAG |  |  |  |  |  |  |
| PCC | -12 | -36 | 38 | 5.98 | 354 | 0.007 FWE |
| **Baseline < Real** |  |  |  |  |  |  |
| *N.S.* |  |  |  |  |  |  |

FC, functional connectivity; VAS, visual analog scale; L, left; R, right; VPL, ventral posterior lateral; IPL, inferior parietal lobule; PAG, periaqueductal gray; PCC, posterior cingulate; MNI, Montreal Neurological Institute

**Table S5:**

Correlations between change in FC and change in clinical pain (VAS) for baseline vs real

| Seed  FC Region | MNI coordinates (x y z) | | | T | Cluster size | Cluster p-value |  |
| --- | --- | --- | --- | --- | --- | --- | --- |
| L S1 |  |  |  |  |  |  | |
| L SMA | -2 | 4 | 52 | 6.98 | 200 | 0.013 FWE | |

FC, functional connectivity; VAS, visual analog scale; L, left; R, right; S1, primary somatosensory cortex; SMA, supplementary motor area; MNI, Montreal Neurological Institute

**Table S6:**

|  | Δ McGill Clinical Pain  Real tDCS - Sham |
| --- | --- |
| L VPL – R posterior insula  Real - Sham FC | r = 0.752  p = 0.008 |
| L VPL – M1/S1  Real - Sham FC | r = 0.602  p = 0.05 |
| L VL – R posterior insula  Real - Sham FC | r = 0.648  p = 0.031 |

FC, functional connectivity; L, left; VPL, ventral posterior lateral; M1, primary motor cortex; S1, primary somatosensory cortex; VL, ventral lateral
